# The exon-skipping oligonucleotide, KitStop, depletes tissue-resident mast cells *in vivo* to ameliorate anaphylaxis

**DOI:** 10.3389/fimmu.2023.1006741

**Published:** 2023-01-31

**Authors:** Barry A. Hedgespeth, Douglas B. Snider, Katie J. Bitting, Glenn Cruse

**Affiliations:** ^1^ Department of Molecular Biomedical Sciences, College of Veterinary Medicine, NC State University, Raleigh, NC, United States; ^2^ Department of Clinical Sciences, College of Veterinary Medicine, NC State University, Raleigh, NC, United States; ^3^ Comparative Medicine Institute, North Carolina State University, Raleigh, NC, United States; ^4^ Comparative Medicine and Translational Research Training Program, North Carolina State University, Raleigh, NC, United States

**Keywords:** mast cell (MC), anaphylaxis, KIT, exon skipping, mast cell depletion

## Abstract

**Introduction:**

Anaphylaxis represents the most extreme and life-threatening form of allergic disease and is considered a medical emergency requiring immediate intervention. Additionally, some people with mastocytosis experience recurrent episodes of anaphylaxis during normal daily activities without exposure to known triggers. While acute therapy consists primarily of epinephrine and supportive care, chronic therapy relies mostly on desensitization and immunotherapy against the offending allergen, which is a time-consuming and sometimes unsuccessful process. These treatments also necessitate identification of the triggering allergen which is not always possible, and thus highlighting a need for alternative treatments for mast cell-mediated diseases.

**Methods:**

The exon-skipping oligonucleotide KitStop was administered to mice intradermally, intraperitoneally, or systemically at a dose of 12.5 mg/kg. Local mast cell numbers were enumerated via peritoneal lavage or skin histology, and passive systemic anaphylaxis was induced to evaluate KitStop’s global systemic effect. A complete blood count and biochemistry panel were performed to assess the risk of acute toxicity following KitStop administration.

**Results:**

Here, we report the use of an exon-skipping oligonucleotide, which we have previously termed KitStop, to safely reduce the severity and duration of the anaphylactic response via mast cell depopulation in tissues. KitStop administration results in the integration of a premature stop codon within the mRNA transcript of the KIT receptor—a receptor tyrosine kinase found primarily on mast cells and whose gain-of-function mutation can lead to systemic mastocytosis. Following either local or systemic KitStop treatment, mice had significantly reduced mast cell numbers in the skin and peritoneum. In addition, KitStop-treated mice experienced a significantly diminished anaphylactic response using a model of passive systemic anaphylaxis when compared with control mice.

**Discussion:**

KitStop treatment results in a significant reduction in systemic mast cell responses, thus offering the potential to serve as a powerful additional treatment modality for patients that suffer from anaphylaxis.

## Introduction

Anaphylaxis is a rapid and potentially fatal systemic allergic reaction that occurs following exposure to an allergen (1). The American College of Allergy, Asthma, and Immunology (ACAAI) anaphylaxis working group estimated the lifetime prevalence of anaphylaxis for a person living in the USA to be up to 5% (2); however, more recent reports indicate that anaphylaxis may be experienced much more frequently than previously reported (3, 4). The most common triggers of anaphylaxis include medications (antibiotics, NSAIDs, antineoplastic/cytotoxic drugs, immunomodulators, contrast dyes), bee and wasp venom, and foods such as peanuts, wheat, milk, and soy (5). Following prior immune sensitization by the allergen, future exposures can result in activation of mast cells (MCs) through IgE- or non-IgE-dependent pathways. Activated MCs release a myriad of inflammatory mediators such as histamine, proteases, cytokines, and growth factors, all of which culminates in the development of clinical signs typically associated with anaphylaxis (6). These include skin flushing, pruritus, angioedema, dyspnea, vomiting, diarrhea, hypotension, hypovolemic shock, and cardiovascular collapse; death may also rapidly occur if the patient is not promptly addressed (7). Treatment relies on the acute mitigation of clinical signs with epinephrine and supportive care followed by chronic strategies such as desensitization and immunotherapy to prevent severe future reactions from occurring (7). These chronic therapies rely on the identification of the allergic trigger which is not always possible; in addition, patients must be willing to attend frequent visits to the clinic and are prone to developing side effects associated with these treatments. Absolute contraindications for some chronic therapies include uncontrolled asthma, atopic dermatitis/eczema, and chronic urticaria, leaving these patients with few other options to address their recurrent anaphylaxis (8). As such, additional therapies for treating anaphylaxis and its sequelae are urgently required for patients who are unable to find recourse in currently available treatments.

While countless triggers can result in varying degrees of anaphylaxis, almost all cases are caused in part by the degranulation of MCs. After exiting the bone marrow, hematopoietic progenitor cells migrate throughout the body and, upon reaching their destination within tissues, develop into mature MCs after receiving the appropriate immunostimulatory signals. Primary destinations for these cells include around blood vessels and nerves, within the skin, and at the mucosal surfaces of the respiratory and gastrointestinal tracts (9). The two primary receptors involved in MC function are the high-affinity immunoglobulin E (IgE) receptor, FcϵRI, and KIT, a receptor tyrosine kinase also known as CD117 (10). While FcϵRI is the primary receptor involved in MC degranulation, KIT is responsible for growth, differentiation, and maturation of MCs *via* binding of its ligand, stem cell factor (SCF) (11). Additionally, the interaction between SCF and KIT are critical for MC proliferation and suppression of apoptosis (12). KIT is initially expressed by most hematopoietic cells but is lost by most during the differentiation process; MCs are one of the few exceptions that retain this receptor for their lifespan (13). Indeed, the gene encoding the KIT protein, *c-Kit*, is considered a proto-oncogene and activating mutations are known to induce the development of cutaneous or systemic mastocytosis—the abnormal accumulation of MCs within tissues (13, 14). Patients suffering from mastocytosis are susceptible to MC activation syndrome (MCAS), a condition characterized by spontaneous, recurrent episodes of systemic anaphylaxis that often do not have a known trigger (15). In addition to antihistamines, treatments aimed at reducing the severity of symptoms experienced by patients suffering from MCAS include glucocorticoids, cytoreductive medications such as interferon-α and cladribine, or tyrosine kinase inhibitors such as masitinib; these are, however, fraught with numerous side effects including cytopenias and increased risk of infections, thereby worsening these patients’ already diminished quality of life (15).

Given its relative scarcity among cell types other than MCs as well as its involvement in numerous debilitating MC-related disorders, KIT provides an alluring therapeutic target for anaphylaxis and MCAS. We have previously shown that MC tumors comprising MCs with gain-of-function KIT mutations can successfully be targeted and significantly reduced by the exon-skipping oligonucleotide (ESO) KitStop (16). ESOs belong to a group of antisense oligonucleotides (ASOs) that block binding of the spliceosome to an intron-exon or exon-intron boundary, thus resulting in the skipping of a particular exon of interest. If the skipped exon maintains the reading frame of the mature mRNA transcript, an alternatively spliced product will be generated. KitStop is a 25-mer morpholino ESO that targets the donor splice site of exon 4 of *c-Kit* pre-mRNA; upon its introduction, KitStop induces a frameshift into the mature mRNA open reading frame, resulting in a premature stop codon. This triggers either the nonsense-mediated decay of the KIT mRNA template or the production of a severely truncated peptide and nonfunctional receptor (16). To determine whether a KIT-specific ESO has any effect on the development and severity of anaphylaxis, here we assessed the impact that KitStop has using a well-established mouse model of passive systemic anaphylaxis, as well as compared its efficacy following varying routes of administration.

## Materials and methods

### Mice

Four to six-week-old female BALB/c mice were purchased from Jackson Laboratories (Bar Harbor, ME) and were maintained at the North Carolina State University laboratory animal facility. Animal care and experimental protocols were conducted with the guidelines of the National Institutes of Health and with the approval of the Institutional Animal Care and Use Committee of North Carolina State University laboratory animal care protocols (17-108-B and 20-223-B).

### ESO design

The KitStop ESO was designed to target exon 4 of murine *c-Kit* (GenBank: NM_001329070.1). A region within the splicing donor site was targeted with the following sequence: 5′-AGGACTTA AACAGCACTCACCTGAG-3′. Specificity of the oligonucleotide sequence was confirmed with BLAST search and was purchased from Gene Tools (Philomath, OR). The unconjugated standard control ASO provided by Gene Tools had the following sequence: 5′-CCTCTTACCTCAGTTACAATTTATA-3′. To facilitate *in vivo* delivery, an octaguanidinium dendrimer was linked to the terminal 3′-N of the standard control and KitStop oligonucleotides (Vivo-Morpholino).

### Peritoneal lavage

Peritoneal lavage fluid was collected as described (17). Mice (7 to 10 weeks old) were euthanized and 5 mL of phosphate-buffered saline (PBS) was instilled into the peritoneal cavity. The abdomen was gently massaged, and the fluid was aspirated from the peritoneum before being placed on ice. The fluid was centrifuged at *300 x g* for 5 minutes, the supernatant was discarded, and the cell pellet was resuspended in PBS for further analysis. A sample was collected for total cell enumeration with Trypan Blue staining, as well as mast cell enumeration with Kimura staining.

### Qualitative reverse transcription-PCR

RNA from cells in the peritoneal lavage fluid was extracted using the RNeasy Plus Mini kit (QIAGEN) according to the manufacturer’s instructions with inclusion of the QIAshredder step. The primers used were designed to amplify exon 4 as well as surrounding exons; their sequences are as follows: forward 50-TCATCGAGTGTGATGGGAAA-30; reverse 50-TCACAGGGGAGATGTTGATG-30. Gel electrophoresis PCR images were acquired using a Licor Odyssey Fc Imaging system.

### Flow cytometry

MCs in peritoneal lavage fluid were identified as double-positive for CD117 and FcϵRIα. Bone marrow-derived mast cells (BMMCs) were treated with 10 µM of either Vivo-Morpholino standard control ASO or Vivo-Morpholino KitStop ESO for 24 hours before flow cytometric analysis. Cells were stained with the following antibodies on ice for 60 minutes before imaging: fluorescein isothiocyanate (FITC)-conjugated rat anti-mouse CD117 (Clone 2B8; BD Pharmingen) and phycoerythrin (PE)-conjugated anti-mouse FcϵRIα (clone MAR-1; eBioscience). Data were acquired using a CytoFLEX (Beckman Coulter) flow cytometer and analyzed with FCS Express version 6 (Denovo Software), or FLOWJO version 10.

### Histologic analysis

Skin biopsies were fixed with 10% neutral buffered formalin at 45°C for 24 hours followed by immersion in 70% ethanol until paraffin embedment. Sections were made every 4 μm and stained with either 0.1% toluidine blue or hematoxylin and eosin (H&E). Images were acquired with an Olympus VS200 whole slide scanner at 40X. Images were -visualized using OlyVIA software from the whole slide images (Olympus Life Science).

### Passive systemic anaphylaxis model

A model of passive systemic anaphylaxis was generated as previously described (18). In brief, 7 to 10 week-old mice were treated intravenously (retro-orbitally) with standard control ASO or KitStop ESO at a dose of 12.5 mg/kg in 100 µl of PBS. One day after the final oligonucleotide injection, mice were sensitized with an intravenous injection of 3 μg mouse anti-2,4-dinitrophenyl (DNP) monoclonal immunoglobulin (Ig) E (clone SPE-7, Sigma-Aldrich). Mice were challenged on the following day with an intravenous injection of 300 μg DNP conjugated to human serum albumin (DNP-HSA, Sigma-Aldrich) in 200 μL PBS. A rectal temperature measurement was obtained immediately prior to and every 10 minutes after DNP-HSA injection for a total of 90 minutes.

### Complete blood count and serum biochemical measurements

Blood was collected from the mice *via* direct cardiac puncture and analyzed at the North Carolina State University Veterinary Clinical Pathology laboratory.

### Beta-hexosaminidase degranulation assay

Bone marrow-derived mast cells (BMMCs) were developed from bone marrow obtained from the femurs of 7-12-week-old C57BL/6J mice (The Jackson Laboratory), as described (17). After being in culture for 4 weeks, 0.5 x 10 (6) BMMCs were treated with 10 µM of either Vivo-Morpholino standard control ASO or Vivo-Morpholino KitStop ESO for 24 hours. The cells were concurrently sensitized with 100 ng/mL anti-DNP IgE (SPE7 clone) (Sigma). After 24 hours, the cells were washed with PBS and used to measure β-hexosaminidase release as previously described (19).

### Statistical analysis

Results represent mean + individual data points or mean ± SEM. An unpaired Student’s t-test was used to evaluate the significance of differences between two datasets. A mixed-effects model (REML) analysis with Sidak’s multiple comparisons test was used to determine statistical significance in comparisons of multiple datasets. Data were considered statistically significant if *p* ≤ 0.05.

## Results

### Local KitStop administration reduces KIT expression and MC number *in vivo*

**Figure 1:**
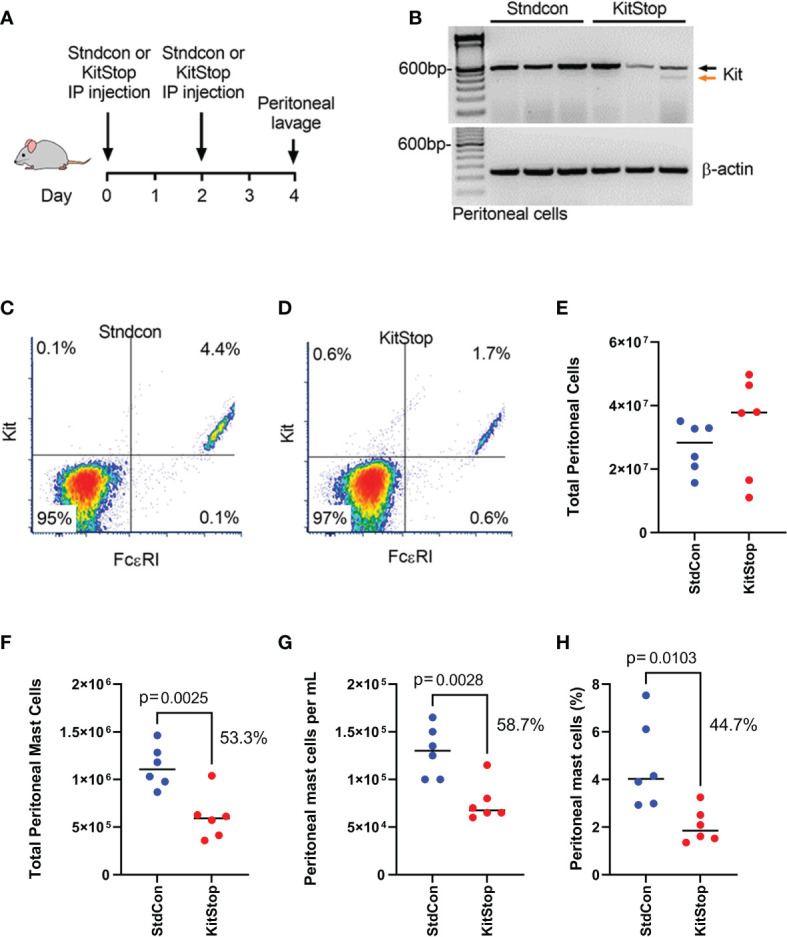
KitStop ESO reduces Kit expression in mouse mast cells locally *in vivo*. **(A)** Balb/c mice received 2 intraperitoneal injections of standard control ASO or KitStop ESO 2 days apart. A peritoneal lavage was performed 2 days after the final injection, and cells in the lavage fluid were analyzed for KIT and FcϵRI expression. **(B)** RT-PCR of c-Kit expression in cells collected by peritoneal lavage (n=3). Black arrow = full-length c-Kit, orange arrow = alternatively spliced c-Kit. **(C, D)** Representative flow cytometry density plots of peritoneal cells harvested from seven mice treated with standard control ASO **(C)** or KitStop ESO **(D)**. **(E)**Total number of cells enumerated in the peritoneal lavage fluid collected. **(F)** Total number of peritoneal mast cells in peritoneal lavage fluid. **(G)** Number of mast cells per milliliter of peritoneal lavage fluid recovered. **(H)** Percent of mast cells out of all cells recovered from the peritoneal lavage fluid. Each datapoint represent a different mouse and data are combined from at least two independent experiments. P value is from an unpaired t-test.

To assess the effect of peritoneal KitStop administration on MC KIT expression as well as total MC number, two intraperitoneal injections of either standard control ASO or KitStop ESO were administered two days apart followed by peritoneal lavage collection two days after the final injection (**Figure 1A**). No overt adverse effects were observed in either group. Two intraperitoneal injections of KitStop appeared to reduce the overall number of transcripts and induced the expression of a truncated splice variant of *c-Kit* in peritoneal MCs whereas no splice variant was expressed in mice treated with the standard control ASO (**Figure 1B**). However, while these data suggested that the KitStop ESO worked in this setting, the qualitative reverse transcription PCR data were not reliable for quantitation and demonstrated some variation in appearance between mice. We have shown that KitStop ESO induces nonsense-mediated decay of the truncated KIT mRNA template, which would diminish the truncated variant with this approach (16). We therefore chose to validate the efficacy of the KitStop ESO with another approach directly measuring MC number. The number of peritoneal MCs (those double positive for FcϵRIα and KIT) decreased by more than half with KitStop treatment when compared to control mice (**Figures 1C, D**). The total number of cells collected from the peritoneal lavage was not different between the treatment groups (**Figure 1E**). However, the total number of MCs, the number of MCs per milliliter of lavage fluid recovered, and overall percentage of peritoneal MCs was significantly lower in the KitStop-treated mice when compared with the standard control ASO treatment (**Figures 1F–H**).

**Figure 2:**
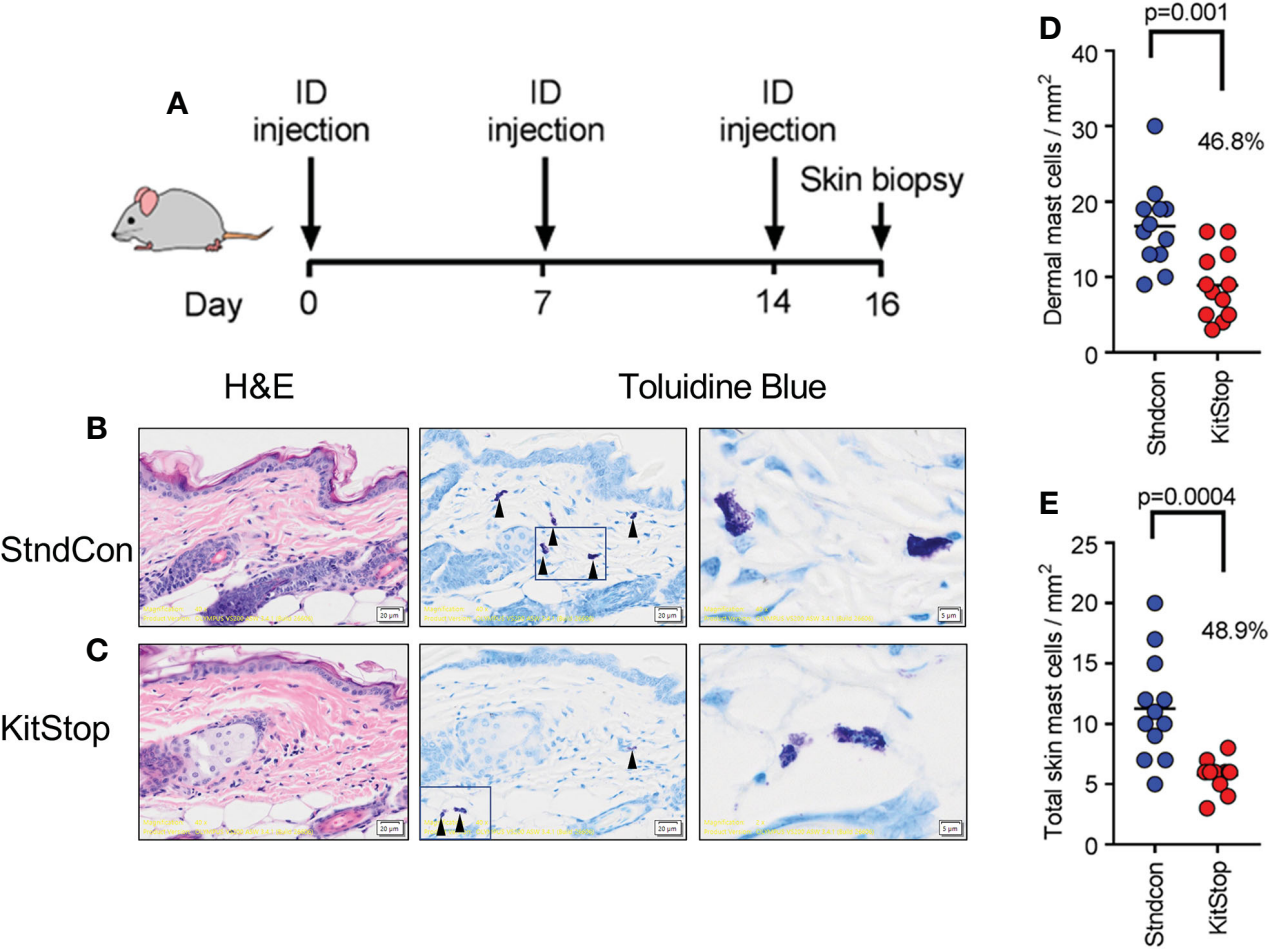
Local KitStop ESO administration reduces mouse dorsal skin mast cell numbers *in vivo*. **(A)** Four Balb/c mice were given dorsal intradermal injections of either standard control ASO or KitStop ESO every 7 days for a total of 3 injections. Skin was then biopsied 2 days after the final injection for histological analysis. **(B, C)** In representative sections from standard control ASO (Stndcon; **B**) and KitStop ESO **(C)** treatment groups, MCs were easily identified due to the presence of positive-staining metachromatic granules, which give a deep violet hue (see red arrow heads). Mast cells displayed normal tissue localization with no evidence of pathology. **(D)** Plot of dermal mast cell number per mm2 from toluidine blue stained skin sections taken from standard control ASO (blue) or KitStop ESO (red) treated skin. **(E)** Plot of mast cell number per mm2 from the full thickness skin sections, including dermis and panniculus adiposus surrounding adnexal structures. Each dot represents the average mast cell number per entire specimen. Three specimens were evaluated from each mouse. P values are from an unpaired t-test.

To evaluate the effect of KitStop on the cutaneous MC population, two intradermal injections of either standard control ASO or KitStop ESO were administered into the dorsal skin of the mice followed by skin biopsy (**Figure 2A**). Histological evaluation of the skin biopsies revealed normal MC tissue localization and no evidence of overt tissue pathology (**Figures 2B, C**). The numbers of MCs per mm (2) in the dermis alone (**Figure 2D**) and full thickness skin sections (**Figure 2E**) were significantly reduced by nearly 50% in the KitStop-treated mice. Taken together, these data indicate that local administration of KitStop significantly reduces MC KIT expression as well as overall MC number.

### Systemic KitStop administration reduces the cutaneous MC population *in vivo*

**Figure 3:**
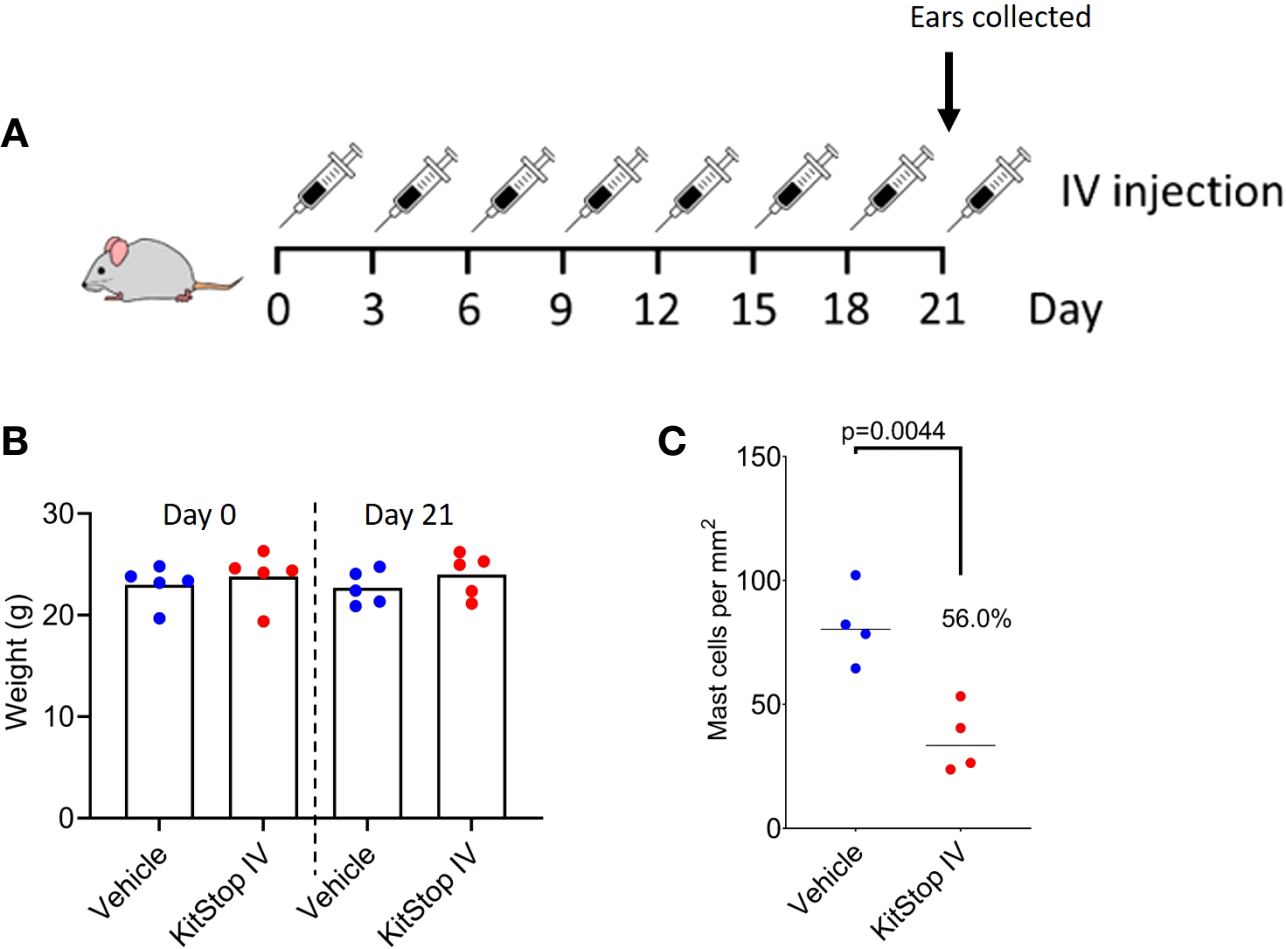
Systemic KitStop ESO treatment reduces the number of cutaneous mast cells in ear skin. **(A)** Five Balb/c mice were treated intravenously with PBS vehicle control or KitStop ESO every 3 days for 21 days. Ears were harvested and evaluated histologically with H&E and toluidine blue stains to facilitate counting of tissue MCs. **(B)** No significant changes in body weight were observed between mice treated with PBS vehicle control (blue) or KitStop ESO (red) at the end of the 21-day treatment period. **(C)** The total number of mast cells per mm2 of ear skin was significantly reduced by 56% in KitStop-treated mice. Data shown are the mean ± SEM for each group. P values are from an unpaired t-test.

We next sought to determine the safety and efficacy of KitStop on MCs when administered systemically. Eight intravenous injections of either PBS vehicle control or KitStop ESO were administered to mice; thereafter, ears were harvested for histological assessment (**Figure 3A**). No adverse effects were observed in either group and no change in body weight occurred for the duration of the treatment course (**Figure 3B**). There was a significant reduction of the aural cutaneous MC population by 56% in KitStop-treated mice following systemic treatment (**Figure 3C**). These data indicate that repeated intravenous KitStop administration does not result in a measurable weight change in mice and produces a significant reduction in the cutaneous MC population.

### KitStop administration significantly reduces the anaphylactic response while having no overt effect on hematopoietic or biochemical parameters over the 21 day protocol

**Figure 4:**
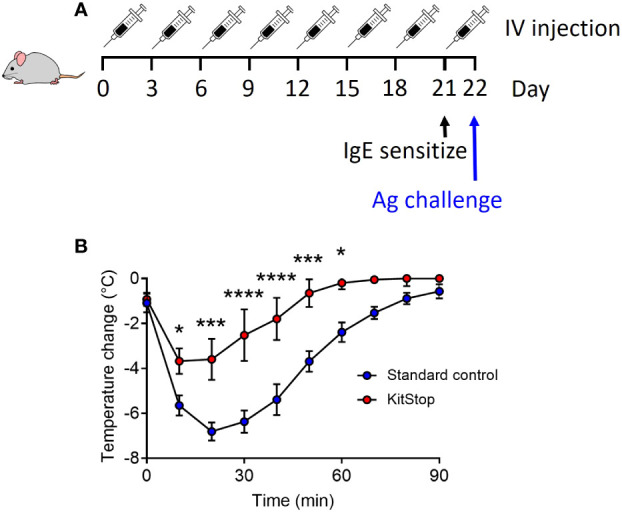
Intravenous KitStop ESO treatment reduces mast cell-mediated passive systemic anaphylaxis. **(A)** A standard control ASO or KitStop ESO was administered intravenously to five Balb/c mice every 3 days for a total of 8 doses. After the last dose, mice were sensitized with an intravenous injection of anti-DNP IgE and challenged the following day with DNP-HSA intravenously. **(B)** After antigen challenge, rectal temperatures of mice treated with either standard control ASO (blue) or KitStop ESO (red) were monitored every 10 minutes for 90 minutes as a surrogate for the measurement of the anaphylactic response. Mice that received the KitStop ESO exhibited a significantly less severe and shorter anaphylactic response than control mice. Data shown are the mean ± SEM for each group of five mice. P values were determined by mixed-effects model (REML) analysis with Sidaks multiple comparisons test. *p ≤ 0.05, ***p ≤ 0.001, ****p ≤ 0.0001.

Having shown that local and systemic KitStop administration is effective at significantly reducing *c-Kit* expression as well as tissue-resident MC numbers, our final aim was to determine KitStop’s global effects in a model of passive systemic anaphylaxis. After receiving 8 intravenous injections of either standard control ASO or KitStop ESO, five mice per group were sensitized with intravenous monoclonal IgE specific for 2,4-dinitrophenyl (DNP). On the following day, the mice were challenged with intravenous DNP-HSA and their rectal temperatures were measured every 10 minutes for a total of 90 minutes (**Figure 4A**). A drop in body temperature of 5-9°C within the first 5-15 minutes is considered a normal systemic anaphylactic response in mice, with recovery to baseline expected to occur within 60-90 minutes (18). In our experiments, KitStop-treated mice experienced a significantly smaller decrease in rectal temperature during the first 10 minutes, with an average reduction of 4°C in contrast to the 6°C decrease experienced by the control mice. This disparity became more significant as time progressed, with a significant difference remaining between the groups until 60 minutes after challenge. Normothermia was achieved more rapidly by KitStop treated mice than control mice, with KitStop treated mice returning to their baseline temperature 30 minutes sooner than control mice (**Figure 4B**). This effect was unlikely attributable to changes in surface expression of the IgE receptor or mast cell degranulation as there was no significant difference in these criteria following treatment of bone marrow-derived mast cells with either Stndcon ASO or KitStop ESO (**Supplemental Figure 1**).

**Figure 5:**
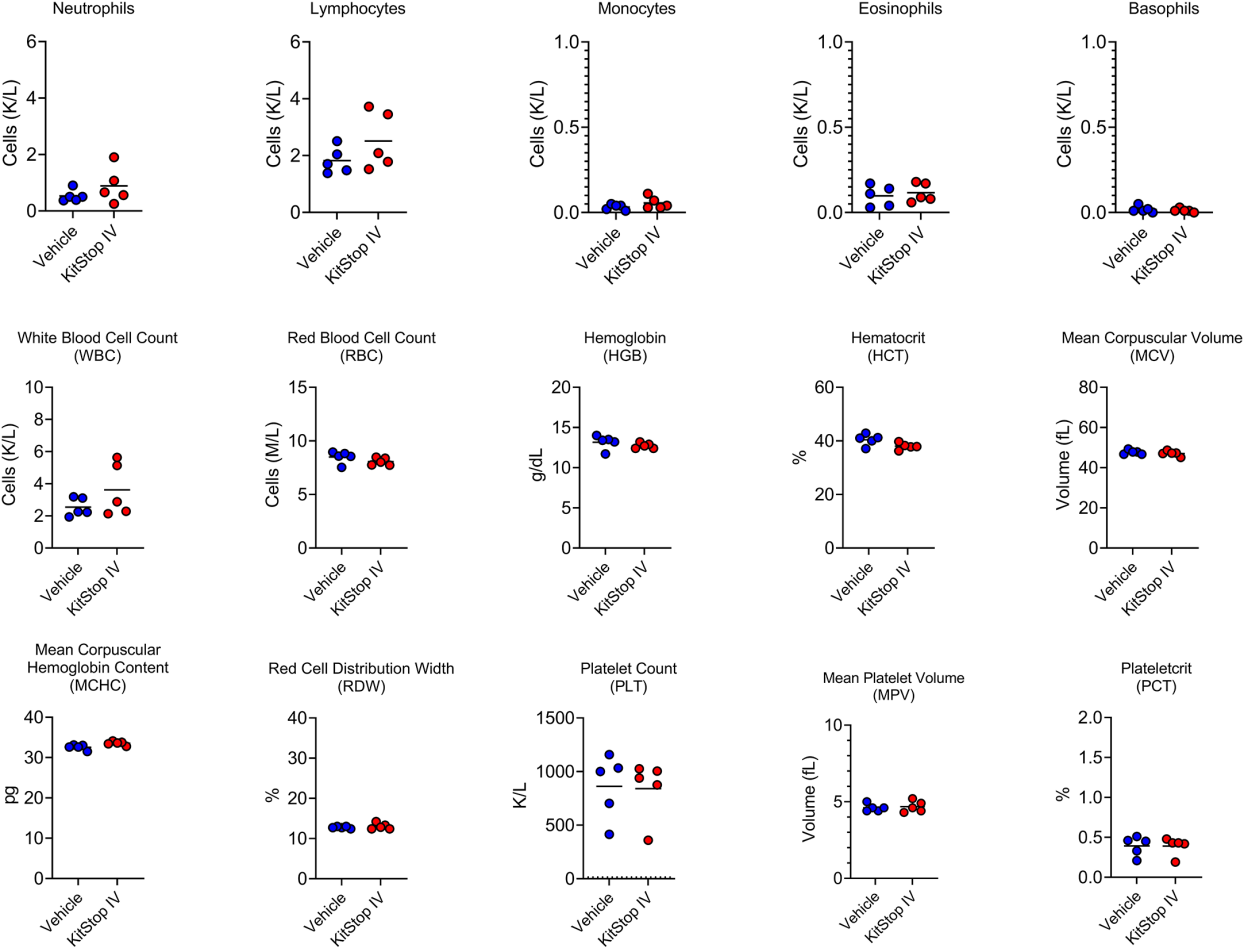
Intravenous KitStop treatment does not result in hematopoietic abnormalities. Complete blood count parameters measured from whole blood isolated from Balb/c mice with cardiac puncture after 21 days of either PBS vehicle control (blue) or KitStop ESO (red) treatment administered every 3 days. No significant differences were observed between the groups.

**Figure 6:**
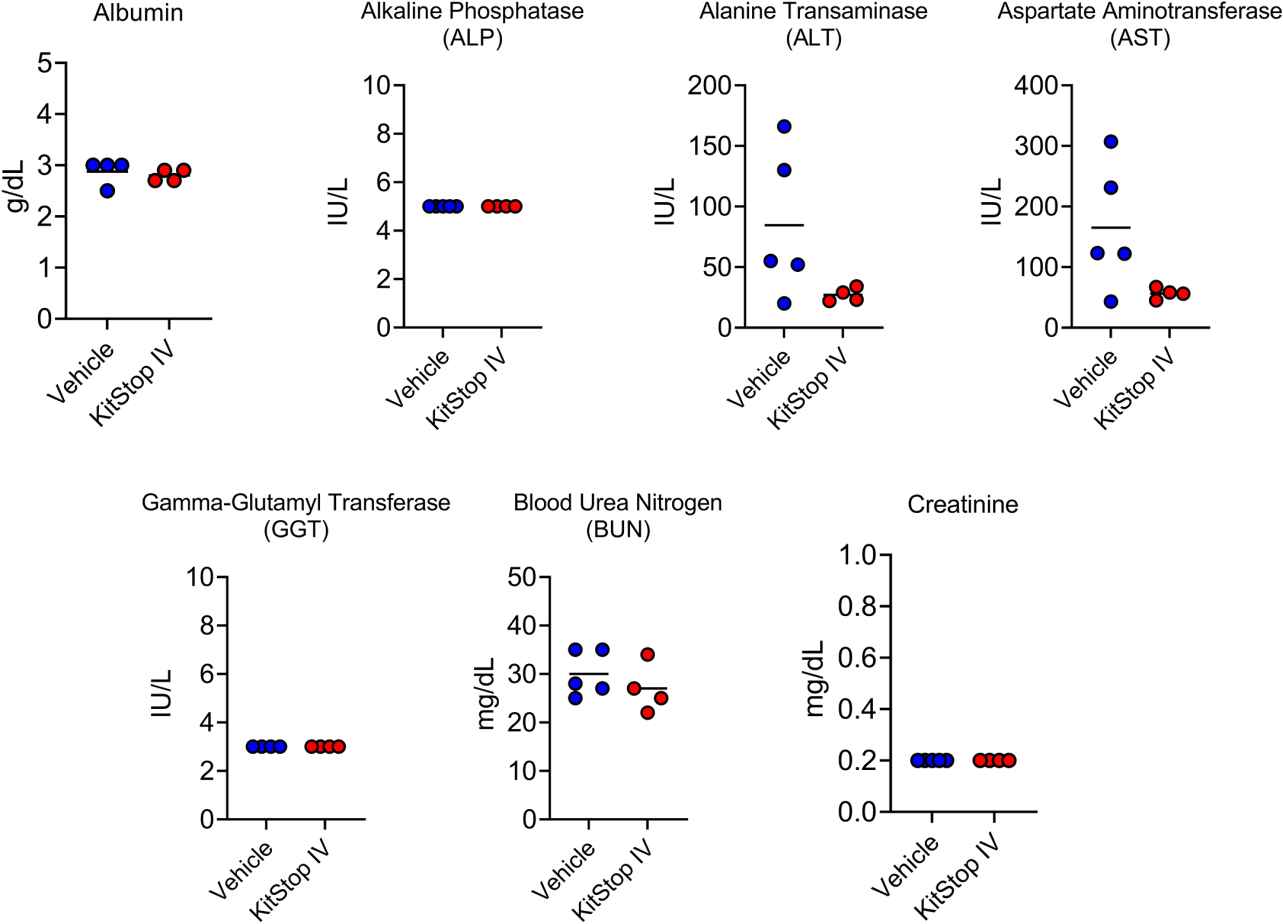
Intravenous KitStop ESO administration does not cause organ dysfunction. Serum biochemical parameters for liver and kidney function measured from whole blood isolated from Balb/c mice with cardiac puncture after 21 days of either PBS vehicle control (blue) or KitStop ESO (red) treatment administered every 3 days. No significant differences were observed between the groups.

Because the KIT receptor is expressed by immature hematopoietic stem cells in addition to MCs, a complete blood count was performed on whole blood from mice that received the same treatment regimen. No differences in erythrocyte, leukocyte, or thrombocyte parameters were noted in either group (**Figure 5**). To assess the effect that oligonucleotide treatment might have on kidney and liver function, biochemistry panels were performed on serum samples from each mouse. No differences in hepatic or renal function were noted for either group (**Figure 6**). Two control mice displayed mildly increased hepatocellular enzyme activities, which was likely due to iatrogenic hepatic trauma during the cardiac puncture procedure. In total, these data indicate that KitStop treatment significantly reduces the severity and duration of the anaphylactic response in a mouse model of passive systemic anaphylaxis, and does not overtly affect hematopoietic stem cell, renal, or hepatic function.

## Discussion

Current therapies for people prone to recurrent anaphylaxis, such as those suffering from MCAS, rely heavily on desensitization, immunotherapy, and a variety of medications. Desensitization and immunotherapy treatments require identification of the triggering allergen; however, it is estimated that 30-60% of patients presenting with anaphylaxis have no discernible trigger (20, 21). If desensitization or immunotherapy is elected, the patient must thereafter be able to attend frequent office visits to carry out treatment which can prove burdensome and sometimes unfeasible. Medications such as glucocorticoids, cytoreductive agents, monoclonal antibodies, and tyrosine kinase inhibitors are commonly used, especially in hospital settings after a patient has experienced an anaphylactic reaction in response to other medications (antibiotics, chemotherapeutics) (22). These medications are fraught with adverse effects such as immunosuppression and gastrointestinal upset, resulting in an increased burden on the patient and healthcare system overall. Moreover, patients with severe, uncontrolled allergic conditions such as asthma or atopy may not even be considered for some of the above-mentioned therapies due to their heightened risk for adverse outcomes (8). Our studies suggest that a noteworthy treatment alternative, KitStop, could be considered for patients suffering from recurrent anaphylaxis who may not be eligible or able to undertake other treatments. We demonstrated that KitStop significantly lowers the number of mature MCs within the skin, and as a result, significantly diminishes the duration and severity of the anaphylactic response. Importantly, KitStop does so regardless of administration route (intradermal, intraperitoneal, or intravenous).

The KIT receptor is expressed on immature hematopoietic stem cells as they exit from the bone marrow which helps to promote their migration, differentiation, and maturation. Binding of SCF to KIT induces homodimerization and trans-phosphorylation of various regions of the receptors; phosphorylated residues then serve as docking sites for signaling molecules, thus propagating the signal downstream (23). Upon maturation, nearly all cells cease to express the receptor, though notable exceptions include MCs, melanocytes, and interstitial cells of Cajal (11). This specificity makes the KIT receptor an enticing therapeutic target; however, off-target effects could be possible given the receptor’s presence on cells other than MCs. Our studies showed that mice treated with KitStop did not experience any significant changes to their hemogram when compared to control mice, suggesting that hematopoietic stem cells of bone marrow origin were not affected by KitStop. Additionally, biochemical parameters relating to kidney and liver function were unaffected by KitStop treatment. Further studies are required to assess the full safety profile of KitStop as the mice in our study were only treated for a maximum of three weeks whereas human patients would be expected to receive treatments for a longer duration.

Splice-switching antisense oligonucleotides (SSOs) are becoming increasingly more prevalent in the field of biomedical research due to their relative specificity and limited adverse effect profile. Numerous mouse models of human disease have been evaluated to determine the efficacy of SSOs for the treatment of genetic neurodegenerative (24–26), neoplastic (27–30), and cardiovascular (31, 32) diseases. In fact, promising human clinical trials utilizing SSOs have also been conducted in people with Duchenne muscular dystrophy (33, 34) as well as spinal muscular atrophy in children (35, 36). While previous studies have used SSOs to target the high-affinity IgE receptor FcϵRI in MCs (19), the present study employs SSO technology to target the KIT receptor in non-transformed cells, providing yet another tool for the treatment of MC-mediated diseases such as anaphylaxis.

Our study is limited in that a model of passive systemic anaphylaxis was utilized; this model relies on the induction of anaphylaxis due to antibodies from passive sensitization rather than antibodies actively produced in response to antigens as in active models. Future studies are needed to ascertain the effect of KitStop in additional models of anaphylaxis. Additionally, mediators of MC degranulation such as proteases were not measured in the serum of the mice undergoing passive systemic anaphylaxis, because of the timing chosen for the experiments and the focus on body temperature responses over 90 minutes. While a decrease in body temperature is a reliable indicator of an anaphylactic response in mice, future studies should include measurements of products of mast cell degranulation at earlier time-points. While no off-target cellular effects were observed in our study, it is possible that these effects could develop with prolonged administration of KitStop further than 21 days. As such, the possible effect of KitStop on hematopoietic cells, as well as other cells expressing Kit such as melanocytes, neural cells, and germ cells, requires additional investigation utilizing long-term administration.

In conclusion, we have shown that intradermal, intraperitoneal, and intravenous administration of the KitStop ESO targeting exon 4 of the *c-Kit* gene results in the rapid and safe depopulation of MCs within tissues. This translates to a lessened systemic anaphylactic response as well as faster resolution of anaphylaxis in general. KitStop could therefore be considered as a feasible treatment alternative for patients experiencing recurrent episodes of anaphylaxis without the side effects of currently administered treatments.

## Data availability statement

The raw data supporting the conclusions of this article will be made available by the authors, without undue reservation.

## Ethics statement

The animal studies were approved by the North Carolina State University Institutional Animal Care and Use Committee.

## Author contributions

BH, DS, and KB participated in the design and execution of experiments and data analysis. BH wrote the manuscript and prepared the figures. GC conceived the project, designed experiments, analyzed data, procured funding and edited the manuscript. All authors contributed to the article and approved the submitted version.
