# Supplementary material for: The exon-skipping oligonucleotide, KitStop, depletes tissue-resident mast cells in vivo to ameliorate anaphylaxis
Source: Front Immunol. 2023 Jan 31;14:1006741. doi: 10.3389/fimmu.2023.1006741 (PMC9927222; doi:10.3389/fimmu.2023.1006741)
Supplement: Supplementary file 1 [file DataSheet_1.docx]

**Supplement**

**The exon-skipping oligonucleotide, KitStop, depletes tissue-resident mast cells *in vivo* to ameliorate anaphylaxis**

Barry A. Hedgespeth^1,2,3,4#^, Douglas B. Snider^1,2,3,4#^, Katie J. Bitting^1^ and Glenn Cruse^1,3^*


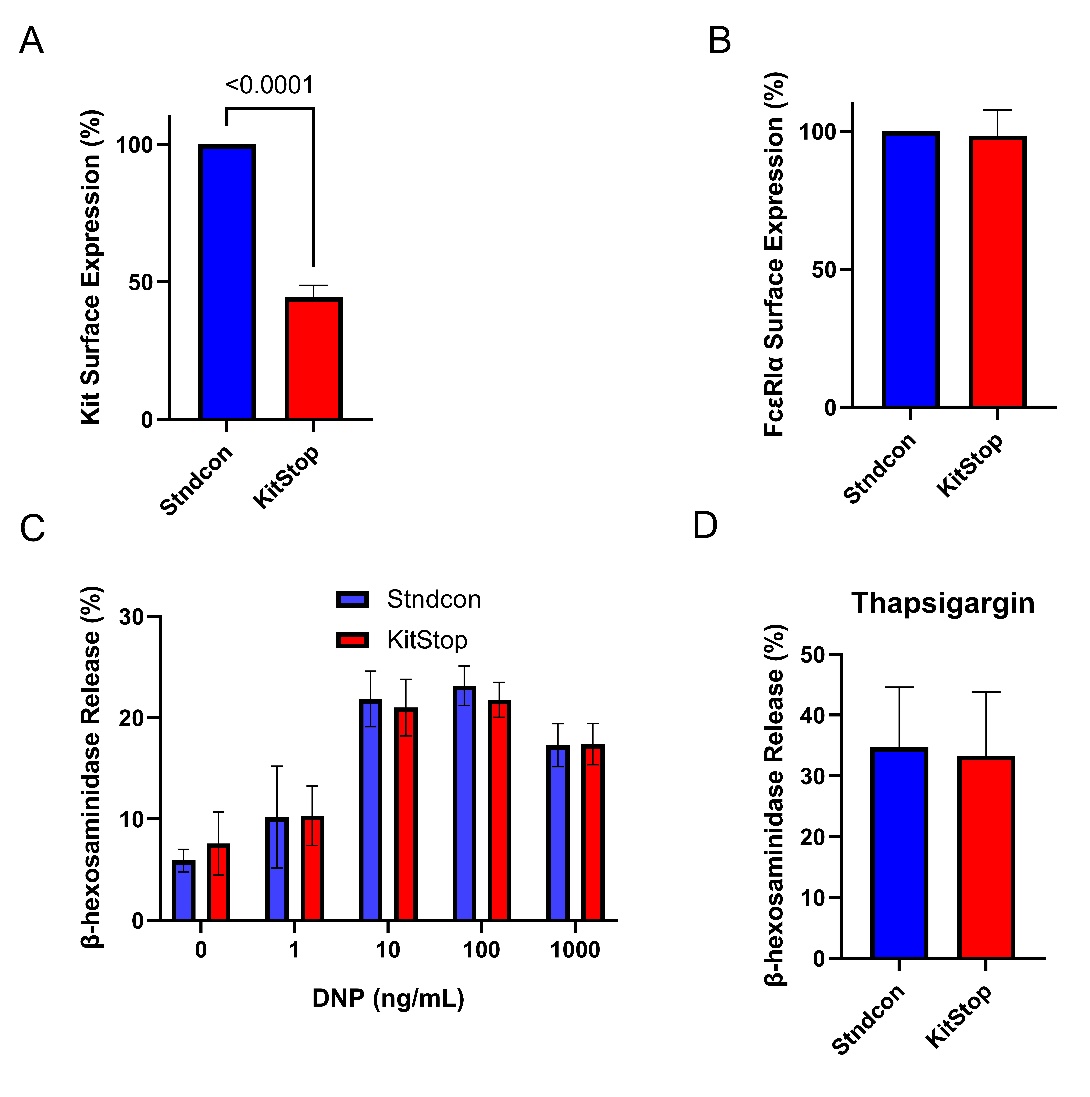


**Supplemental Figure 1: KitStop ESO administration does not affect FcεRIα surface expression or in vitro degranulation.** (A) The surface expression of c-Kit was significantly reduced in murine bone marrow-derived mast cells (BMMCs) treated with 10 μM KitStop ESO when compared to those treated with standard control ASO (StndCon). (B) No difference in surface expression of FcεRIα was observed following treatment with either standard control ASO or KitStop ESO. (C-D) No difference in IgE-mediated or non-IgE-mediated degranulation was observed in BMMCs following treatment with Stndcon or KitStop.
